# Optimal Time Between Completion of Preoperative Chemotherapy and Surgery for Locally Advanced Pancreatic Cancer

**DOI:** 10.1245/s10434-026-19264-2

**Published:** 2026-03-03

**Authors:** Marionna Cathomas, Christoph Kahlert, Thomas Hank, Ingmar Rompen, Maximilian Kryschi, Ulf Hinz, Thilo Hackert, Mohammed Al-Saeedi, Markus W. Büchler, Martin Loos

**Affiliations:** 1 Department of General, Visceral and Transplant Surgery, Heidelberg University Hospital, Heidelberg, Germany; 2 Department of General, Visceral and Thoracic Surgery, University Hospital Hamburg-Eppendorf, Hamburg, Germany; 3 Botton-Champalimaud Pancreatic Cancer Center, Champalimaud Foundation, Lisbon, Portugal

**Keywords:** Pancreatic cancer, PDAC, Surgical resection, Chemotherapy, NAT, Timing, Prognosis

## Abstract

**Background:**

Multimodal treatment with preoperative chemotherapy is increasingly used for locally advanced pancreatic cancer (LAPC) to enable surgical resection. However, the optimal time between the last cycle of preoperative chemotherapy and surgery remains unclear. This study aimed to evaluate the association between timing of surgery after preoperative chemotherapy and overall survival of patients with LAPC.

**Methods:**

Patients with LAPC who underwent pancreatic resection after preoperative chemotherapy between 2018 and 2023 were enrolled from a prospectively maintained database. The cohort was stratified by a predefined interval between the last cycle of chemotherapy and surgery (short interval: < 4 weeks vs long interval: ≥ 4 weeks).

**Results:**

After preoperative chemotherapy, 169 patients underwent surgery, including 56 (33.1%) patients in the short-interval group and 113 (66.9%) patients in the long-interval group. Baseline characteristics, surgical procedures, and postoperative morbidity were comparable between the two groups. Most patients received FOLFIRINOX (85.5 %) with a median of nine cycles. Patients with the long interval showed significantly improved overall survival from the time of LAPC diagnosis (20.7 vs 29.5 months; *p *= 0.019) as well as from surgery (16.1 vs 23.1 months; *p *= 0.024). In multivariable analysis, the interval of 4 weeks or longer was an independent predictor of improved survival. Further analysis suggested an optimal time window of 4–8 weeks between the last chemotherapy cycle and surgery.

**Conclusion:**

An interval of at least 4 weeks between the last cycle of preoperative chemotherapy and surgery for LAPC was associated with significantly improved overall survival, with an optimal window of 4–8 weeks.

**Supplementary Information:**

The online version contains supplementary material available at 10.1245/s10434-026-19264-2.

Pancreatic ductal adenocarcinoma (PDAC) has a dismal prognosis, largely due to late presentation and aggressive tumor biology.^1^^–^^3^ Even in the absence of distant metastases, more advanced tumors are frequently deemed unresectable as a result of vascular involvement.^4^ During the past two decades, multimodal treatment strategies with modern multi-agent chemotherapy have gained wide acceptance. Nevertheless, complete surgical resection remains the most important determinant of survival in PDAC.^5^^–^^7^

In locally advanced pancreatic cancer (LAPC), preoperative chemotherapy is the standard initial approach, aiming to control micrometastatic disease, achieve tumor downstaging, and ultimately enable margin negative resection.^8^ Although the oncologic benefit of preoperative chemotherapy is well recognized, no clear guidelines exist regarding the optimal sequencing and timing of surgery after preoperative chemotherapy, resulting in heterogeneous practice patterns.^9^

Reported resection rates can exceed 60%, particularly after preoprative chemotherapy with FOLFIRINOX, but outcomes may be further improved by refining surgical timing.^10^ Current recommendations are inconsistent and largely based on expert opinion rather than high-level evidence.^11^ Data from other malignancies, such as rectal cancer, indicate that the timing of surgery after systemic therapy can significantly affect oncologic outcomes.^12^^,^^13^ Defining the optimal window for resection after preoperative chemotherapy in LAPC is, therefore, essential to maximize survival benefit while minimizing perioperative morbidity.

This study aimed to evaluate overall survival according to the interval between the last cycle of preoperative chemotherapy and surgery for patients with LAPC.

## Methods

### Study Design and Population

This retrospective study enrolled patients undergoing oncologic pancreatic resection for LAPC after preoperative chemotherapy between January 2018 and June 2023 at the Department of Surgery, Heidelberg University Hospital, Germany. Demographic and clinical data were retrieved from the prospectively maintained institutional database. The local Ethics Committee of the University Heidelberg, Germany approved the study (S-832/2024).

**Fig. 1:**
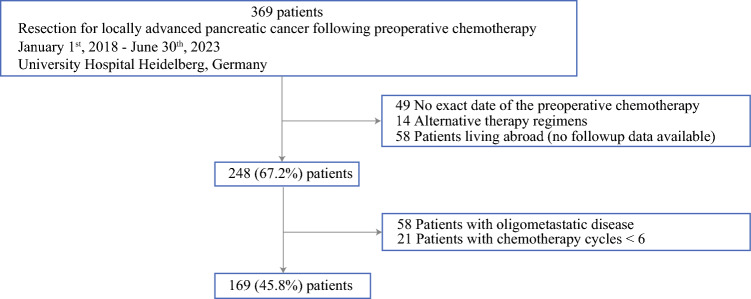
Flow chart showing the number of including patients before and after exclusion. Preoperative chemotherapy: i) FOLFIRINOX (fluorouracil, leucovorin, oxaliplatin and irinotecan), ii) gemcitabine plus nanoparticle albumin-bound (nab)–paclitaxel and iii) switch on second line therapy

Patients were eligible for inclusion in the study if they were at least 18 years of age, had a preoperatively histologically or cytologically proven PDAC, and had undergone either FOLFIRINOX or a gemcitabine-based chemotherapeutic regimen. The exclusion criteria ruled out distant metastasis, alternative therapy regimens, fewer than six cycles of preoperative chemotherapy, no exact date of the last cycle of preoperative chemotherapy, and incomplete follow-up evaluation. The study also excluded patients with primary radiotherapy (Fig. 1).

### Radiologic Assessment and Oncologic Treatment

Radiologic diagnosis of LAPC was adapted from the recommendation of the National Comprehensive Cancer Network (NCCN).^3^ Shortly, if venous involvement of the tumor is technically non-amenable to reconstruction or tumor contact greater than 180° with the superior mesenteric artery or celiac axis.^8^

Preoperative chemotherapy consisted of at least six cycles of FOLFIRINOX (fluorouracil, leucovorin, oxaliplatin and irinotecan), gemcitabine plus nanoparticle albumin-bound (nab)–paclitaxel, and the switch on the others therapy regimen. Cross-sectional imaging-based restaging was performed after every 3 months of treatment. With achievement of resectability criteria on the cross-sectional imaging with stable or decreasing carbohydrate antigen 19-9 (CA 19-9), the indication for open exploration and surgical resection was made.^14^ After conversion surgery, additional adjuvant chemotherapy was generally recommended for patients with fewer than 12 cycles of preoperative chemotherapy. Patients with 12 cycles of preoperative chemotherapy completed were referred for routine oncologic surveillance.

### Follow-up Evaluation and Clinical Variables

The primary endpoint of this study was evaluation of overall survival depending on the short- and long-term interval between the last cycle of preoperative chemotherapy and surgery. The secondary endpoint was definition of an optimal time window within the interval. Predictive factors for reaching significant longer overall survival were elucidated in a multivariable analysis.

Medical or surgical conditions deviating from the normal postoperative course were considered as complications and subdivided according to the Clavien-Dindo classification from 1 to 5, with ≥ 3b considered to indicate a major complication.^15^ All complications, including re-operations and re-admissions, were considered when they occurred within 30 days after discharge of the patient.

Patients were followed up until their latest surveillance or death. Update of follow-up evaluation was performed until 30 June 2023 through medical records or Krebsregister Baden-Württemberg.

### Statistical Analysis

Statistical analyses were performed using the statistical program SPSS Statistics 3.0. Descriptive and univariate analyses were performed by Student’s *t* test and the Mann-Whitney *U* test for continuous variables with normal and non-normal distribution, respectively. Either the median or mean was given in each case. For nominal variables, the chi-square test was applied. The Kaplan-Meier curve (log-rank test) was used to compare overall survival, with overall survival excluded for patients with a grade 5 complication according to Clavien-Dindo classification.

To determine the threshold value for short- and long-term intervals, patients were divided into groups according to a 1-week interval. Afterward, their median overall survival was compared for evaluation of a significant change. For multivariable analysis, the Cox regression model was used to predict independent factors for overall survival. The results were expressed as hazard ratios (HRs), with an HR greater than 1 considered as a positive predictive factor for better overall survival.

Analysis using the Cox proportional hazards model was performed to determine the optimal time window. For this analysis, the overall median survival of the entire study cohort was used as the reference (HR, 1). In the restricted cubic spline curve, an HR lower than 1 was associated with a better overall survival. The vertical line indicates the days at which the curve cross HR was 1. For all analysis, a two-sided *p* value of 0.05 or lower was taken to indicate statistical significance.

## Results

### Patient Cohort and Characteristics

During the study period, 369 patients underwent pancreatic resection after preoperative chemotherapy, 200 (54.2%) of whom were excluded according to predefined criteria (Fig. 1). The final study population comprised 169 patients (45.8%) with a mean age of 62.6 years (range, 39.5–84 years) and a nearly equal sex distribution (51.5% male, 48.5% female). Most tumors were located in the pancreatic head (56.8%), and the median preoperative CA19-9 level was 38.4 U/mL (range, 0.9–6472 U/mL).

The majority of the patients received either FOLFIRINOX (85.5%) followed by a switch of therapy regimen (7.7%) and gemcitabine/nab-paclitaxel alone (6.5%). The median number of chemotherapy cycles for the entire cohort was 9 (range, 6–28; Table 1) The most common surgical procedure was partial pancreatoduodenectomy (40.8%), followed by total pancreatoduodenectomy (32.0%). Major postoperative complications occurred for 26 patients (15.4%; Table 2). Adjuvant chemotherapy data were available for 150 patients (88.8%), 101 (67.3%) of whom received systemic therapy after surgery. The median follow-up period for the entire cohort was 27 months (range, 9.1–87.6 months), with a 90-day mortality of 3.6%.

**Table 1:** Patients’ characteristics of the cohort

	All patients (n = 169)	Short-term interval (n = 56)	Long-term intervall (n = 113)	*p*-value
Age (mean)	62.6 (39.5–84)	62.7 (42.5–78)	62.5 (39.5–84)	0.9
Gender (male)	87 (51.5%)	29 (51.8%)	58 (51.3%)	1.0
BMI (median)	24.0(15.6–36.8)	23.4(18.5–35.8)	24.4(15.6–36.8)	0.2
ASA				0.7
1 + 2	84(49.7%)	29 (51.8%)	55 (48.7%)	
3 + 4	85 (50.3%)	27 (48.2%)	58 (51.3%)	
Localisation of the tumor				0.8
Head	96 (56.8%)	31 (55.4%)	65 (57.5%)	
BodyHail	73 (43.2%)	25 (44.6%)	48 (42.5%)	
Tumour marker				
CA 19-9 (median)	38.4 (0.9–6472)	43.1 (0.9–6472)	37.0 (0.9–3536.4)	0.2
CEA (median)	1.9 (0.1–21.2)	1.7 (0.1–21.2)	1.7 (0.1–17.7)	0.3
Preoperative chemotherapy				1.0
FOLFIRINOX only	145 (85.7%)	48 (85.8%)	97 (85.8%)	
Gemcitabine/nab-paclitaxel only	11 (6.5%)	4 (7.1%)	7 (6.2%)	
Switch on second line therapy	13 (7.7%)	4 (7.1%)	9 (8.0%)	
Preoperative chemotherapy cycles (median)	9 (6–28)	9 (6–28)	10 (6–20)	0.4
Adjuvant chemotherapy	n = 150	n = 49	n = 101	0.7
No	49 (32.7%)	12 (24.5%)	27 (26.7%)	
Yes	101 (67.3%)	37 (75.5%)	74 (73.3%)	
Tumor stage				0.1
O + l	49 (29.0%)	15 (26.8%)	34 (30.1%)	
II	70 (41.1%)	19 (33.9%)	51 (45.1%)	
III	50 (29.6%)	22 (39.3%)	28 (24.8%)	
Resection margin				0.8
RO	110 (65.1%)	43 (76.8%)	67 (59.3%)	
R1	53 (31.3%)	12 (21.4%)	41 (36.3%)	
RX	6 (3.6%)	1 (1.8%)	5 (4.4%)	

*BMI*, body mass index in kg/m2; *ASA*, Grading in American Society of Anesthiologists; CA 19-9, carbohydrate antigen 19-9; *CEA*, carcinoembryonic antigen

**Table 2:** Surgical and clinical outcome

	All Patients (n = 169)	Short-term interval (n = 56)	Long-term intervall (n = 113)	*p*-value
Oncological pancreas resection				1.0
Partial Pancreaticoduodenectomy	69 (40.8%)	22 (39.3%)	47 (41.6%)	
Distal Pankreatectomy	46 (27.2%)	16 (28.6%)	30 (26.5%)	
Total Pancreaticododenectomy	54 (32.0%)	18 (32.1%)	36 (31.9%)	
Vascular resection				0.3
No	47 (27.8%)	13 (23.2%)	34 (30.1%)	
Yes	122 (72.2%)	43 (76.8%)	79 (69.9%)	
Venous resection				0.9
No	68 (40.2%)	23 (41.1%)	45 (39.8%)	
Yes	101 (59.8%)	33 (58.9%)	68 (60.2%)	
Arterial resection				0.3
No	121 (71.6%)	37 (66.1%)	84 (74.3%)	
Yes	48 (28.4%)	19 (33.9%)	29 (35.7%)	
Multivisceral resection				0.3
No	118 (69.8%)	36 (64.3%)	82 (72.6%)	
Yes	51 (30.2%)	20 (35.7%)	31 (27.4%)	
Severity of complication*				0.9
No complication	87 (51.5%)	28 (50.0%)	59 (52.2%)	
< 3a	56 (33.1%)	20 (35.7%)	36 (31.9%)	
> 3a	26 (15.4%)	8 (14.3%)	18 (15.9%)	
90-day mortality				1.0
No	163 (96.4%)	54 (96.4%)	109 (96.4%)	
Yes	6 (3.6%)	2 (3.6%)	4 (3.6%)	
Recurrence	n = 159	n = 53	n = 106	0.4
None	8 (5.0%)	1 (1.9%)	7 (6.6%)	
Local	27 (18.0%)	10(18.9%)	17 (16.1%)	
Distant	50 (31.5%)	17 (32.1%)	33 (31.1%)	
Both	21 (13.2%)	5 (9.4%)	16 (15.1%)	
Not available	53 (33.3%)	20 (37.7%)	33 (31.1%)	

^*^According to Clavien-Dindo Classification within 30 days after discharge

### Threshold and Group Characteristics

**Fig. 2:**
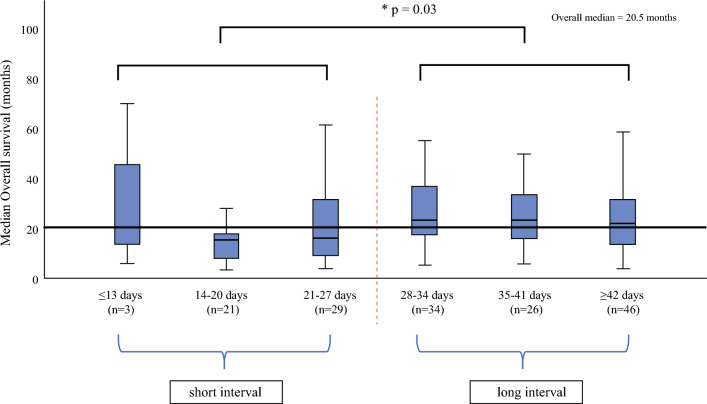
Defining short-term interval and long-term interval between last cycle of preoperative chemotherapy and conversion surgery. All patients were grouped according to one-week time interval between last cycle of preoperative chemotherapy and surgery. A difference could be observed between 4 weeks.

A significant decline in the median overall survival (*p *= 0.03) could be found at 4 weeks. Therefore, < 4 weeks and ≥ 4 weeks between the last preoperative chemotherapy cycle and surgery were defined as the thresholds for short- and long-term interval, respectively (Fig. 2). According to this threshold, 56 patients (33.1%) were classified into the short-interval group and 113 (66.9%) into the long-interval group.

Baseline characteristics, tumor-specific data, and the type and extent of resection were comparable between the two groups. Most of the patients in both groups received FOLFIRINOX (85.7% vs 85.8%), with a median of 9 cycles (range, 6–28 cycles) in the short-interval group versus 10 cycles (range, 6–20 cycles) in the long-interval group (Table 1). Major complications (≥ 3b) occurred for 8 patients (14.3%) in the short-interval group and 18 patients (15.9%) in the long-interval group (Table 2).

### Oncologic Outcome

**Fig. 3:**
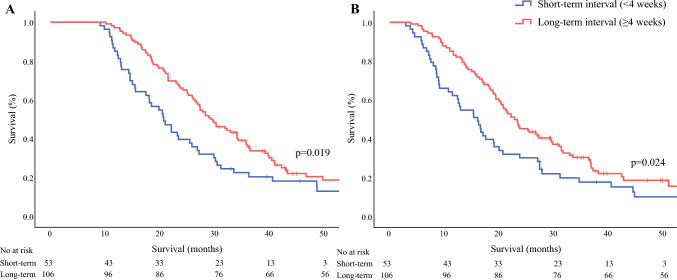
Kaplan-Meier Curves showing overall survival from first diagnosis (**A**) and from surgery (**B**) of patients with short-term and long-term time interval

The median overall survival from the time of LAPC diagnosis was significantly longer in the long-interval group (20.7 vs 29.5 months; *p *= 0.019), as well as the median overall survival from surgery (16.1 vs 23.1 months; *p *= 0.024; Fig. 3). In the short-interval group, the 1-year survival rates were 62.3% compared with 83.0% in the long-interval group (*p *= 0.004). Data on recurrence were available for 106 (66.7%) of all the patients. No difference was found in the recurrence rate (90.4% vs 96.7%; *p *= 0.430). Restricted cubic spline curve further indicated an optimal time window of 4–8 weeks between the last chemotherapy cycle and surgery (Fig. S1).

Across the entire cohort, worse survival was observed among the patients with a higher tumor stage, a short interval between preoperative chemotherapy and surgery, or absence of adjuvant chemotherapy (Table S1). In multivariable analysis, an interval of ≥ 4 weeks and receipt of adjuvant chemotherapy were independent predictors of improved overall survival (Table 3).

**Table 3:** Multivariable analysis to investigate independent factors for overall survival

	HR	Lower.CI	Upper.CI	*p*-value
Age (< 75 years: > 75 years)	1.8	0.8	3.7	0.1
Sex (female:male)	1.1	0.7	1.6	0.6
Tumour stage (0/1:2/3)	2.2	1.0	5.2	0.06
Lymphnode stage (0:1/2)	0.6	0.3	1.3	0.2
Complications (<3a:>3a)	1.4	0.8	2.5	0.303
Time interval (< 4 weeks: > 4 weeks)	0.6	0.4	1.0	0.028
Adjuvant chemotherapy (yes:no)	2.8	1.7	4.6	<0.001

Positive predicitve factors were time interval > 4 weeks and receipt of adjuvant chemotherapy

## Discussion

This study investigated the optimal time between the last cycle of preoperative chemotherapy and surgery for patients with LAPC. Patients who underwent surgery 4 weeks or longer after completion of chemotherapy had significantly longer overall survival, with a suggested optimal window of 4–8 weeks. Importantly, clinicopathologic features and postoperative outcomes were comparable between the two groups, supporting the validity of the survival difference. Multivariable analysis confirmed an interval of 4 weeks or longer as an independent predictor of improved survival, together with receipt of adjuvant chemotherapy.

The results align with evidence from other malignancies. In rectal cancer, current evidence recommends that surgery be delayed beyond 4–6 weeks after neoadjuvant therapy because timing has been shown to influence oncologic outcomes.^16^^,^^17^ For LAPC, recommendations remain inconsistent. A Delphi-based consensus proposed resection within 2–6 weeks, but this result was based on expert opinion in the absence of robust data.^11^ The current study therefore provides real-world evidence that a longer interval (> 4 weeks) may be beneficial for patients with LAPC. The analysis of the optimal time interval showed a survival benefit for patients even beyond 8 weeks. However, it must be assumed that the effect of the chemotherapy diminishes at some point. Therefore, an optimal window of 4–8 weeks is suggested.

A median overall survival of 28.3 months for patients resected after 4 weeks or longer is favorable and lies at the upper range of outcomes reported in contemporary LAPC series after FOLFIRINOX-based chemotherapy, with median survival typically ranging between 20 and 30 months, supporting the external validity of our findings.^10^^,^^18^ However, although most prior studies have focused on conversion rates and regimen efficacy, few have systematically addressed surgical timing as a prognostic factor. Our analysis therefore provides new evidence that the interval between primary chemotherapy and surgery itself may influence survival.

From a biologic perspective, the therapeutic effect of chemotherapy is thought to occur not immediately, but increasing over weeks based on a delayed tumor cell apoptosis. Tumor cell apoptosis, however, leads to activation of the immunologic response, potentiating the effect of chemotherapy.^19^ This remodeling requires more time, supporting a delayed surgical approach.^20^ However, prolonged intervals may increase the risk of fibrosis or tumor regrowth once chemotherapy efficacy wanes.^21^ In the current cohort, no differences in complication rates or pathologic tumor stages were observed between groups, arguing against a major disadvantage of longer intervals.

Several limitations of this study must be acknowledged. More than half of the initially eligible patients were excluded, mainly due to missing data on the exact completion date of preoperative chemotherapy. This high exclusion rate posed a risk of bias. However, precise determination of the end date of preoperative chemotherapy was considered essential for evaluating the impact of the time interval.

Furthermore, regarding the retrospective design of the study, only the number of preoperative chemotherapy cycles was available, without information on dosage and dosage reductions, although dose-based metrics would be preferable in a cohort comprising different regimens. During the study period, no systematic record of tumor regression was performed. Therefore, no conclusion about the impact of a prolonged time interval on tumor regression can be made. The exact mechanisms underlying the observed benefit of a longer interval remain speculative. A possible explanation could be an improved recovery from chemotherapy-related toxicity or optimization of nutritional and functional status before surgery. Furthermore, early progression for patients with a longer interval could be detected during restaging. These patients may have been excluded from surgery, but these data were not available.

## Conclusion

This study demonstrated that for patients with LAPC, an interval of at least 4 weeks between the last cycle of preoperative chemotherapy and surgery was associated with improved survival without increased perioperative risk for complications. Multivariable and restricted spline analyses identified an optimal window of 4–8 weeks. These findings provide real-world evidence to guide clinical decision-making and highlight surgical timing as a potentially modifiable factor to improve outcomes in LAPC.

## Supplementary Information

Below is the link to the electronic supplementary material.

Supplementary file1 (PDF 24 KB)

Supplementary file2 (PDF 95 KB)
