# Supplementary material for: Optimal Time Between Completion of Preoperative Chemotherapy and Surgery for Locally Advanced Pancreatic Cancer
Source: Ann Surg Oncol. 2026 Mar 3;33(6):5060–7. doi: 10.1245/s10434-026-19264-2 (PMC13179189; doi:10.1245/s10434-026-19264-2)

**Supplementary data, Figure 1:** Restricted cubic-spine cox model for optimal time window for surgery after last cycle of preoperative chemotherapy.

Optimal time window for surgery lies betwen 4 and 8 weeks after last cycle of preoperative chemotherapy.

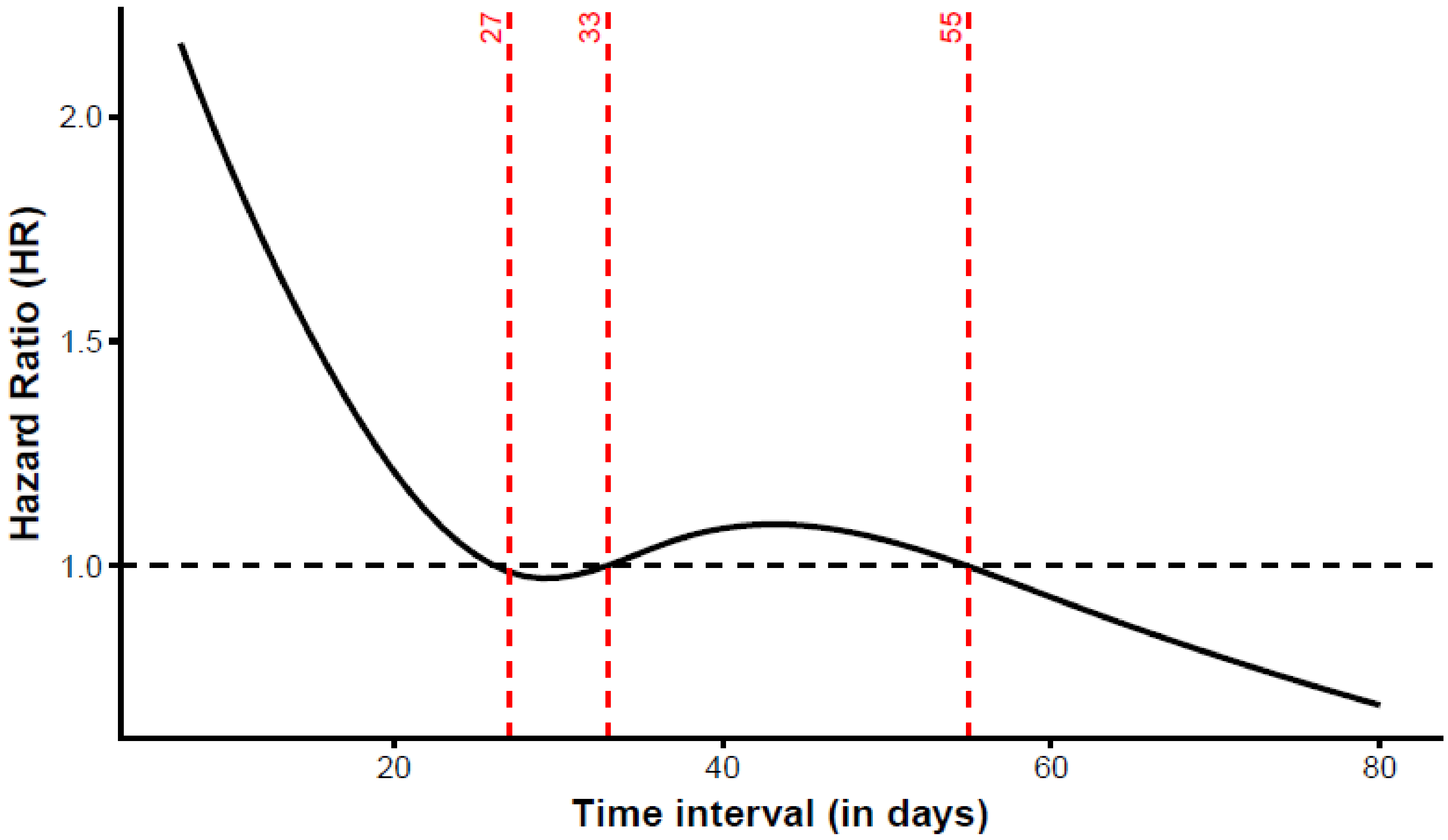

Supplement: Supplementary file 1 — Supplementary file1 (PDF 24 KB) [file 10434_2026_19264_MOESM1_ESM.pdf]
