# Supplementary material for: Optimal Time Between Completion of Preoperative Chemotherapy and Surgery for Locally Advanced Pancreatic Cancer
Source: Ann Surg Oncol. 2026 Mar 3;33(6):5060–7. doi: 10.1245/s10434-026-19264-2 (PMC13179189; doi:10.1245/s10434-026-19264-2)
Supplement: Supplementary file 2 — Supplementary file2 (PDF 95 KB) [file 10434_2026_19264_MOESM2_ESM.pdf]

Supplementary data, Table 1: Association of clinical factors with overall survial.

BMI, body mass index in kg/m^2; ASA, Grading in American Society of Anesthiologists;
CA 19-9, carbohydrate antigen 19-9

	Overall survival	Lower.CI	Upper.CI	p-value
**Age**				**0.8**
≤ 75 years	20.5	17.9	23.0	
> 75 years	20.0	6.8	33.5	
**Gender**				**0.5**
Male	23.4	18.3	28.5	
Female	19.1	15.9	22.2	
**BMI**				**0.5**
≤ 25 kg/m2	20.0	17.1	22.9	
> 25 kg/m2	21.2	16.1	26.3	
**CA 19-9**				**0.2**
≤500	20.8	17.8	23.8	
> 500	15.1	11.4	18.8	
**Time interval**				**0.024**
< 4 weeks	16.1	12.2	20.1	
≥ 4 weeks	22.9	19.1	26.7	
**Adjuvant chemotherapy (n=140)**				**<0.001**
no	9.7	6.4	13.0	
yes	23.8	19.0	29.0	
**Tumour stage**				**0.031**
0 + I	23.8	20.0	27.6	
II	20.0	16.8	23.2	
III	17.0	13.9	20.0	
**Lymphnode stage**				**0.1**
0	23.7	19.6	27.7	
1	20.8	17.7	24.0	
2	17.0	14.1	19.8	
